# Success Rate of Immediately Loaded Orthodontic Temporary Anchorage Devices: A Retrospective Analysis of Patient- and Treatment-Related Factors

**DOI:** 10.3390/bioengineering13070790

**Published:** 2026-07-09

**Authors:** Krisztina Martha, Silvia Izabella Pop, Lenard Farczadi, Radu Vasile Pop, Csaba Dudás, Kinga Mária Jánosi

**Affiliations:** 1 Faculty of Dental Medicine, George Emil Palade University of Medicine, Pharmacy, Science and Technology of Târgu Mureș, 540139 Târgu Mureș, Romania; krisztina.martha@umfst.ro (K.M.); kinga.janosi@umfst.ro (K.M.J.); 2 Chromatography and Mass Spectrometry Laboratory, Center for Advanced Medical and Pharmaceutical Research, George Emil Palade University of Medicine, Pharmacy, Science and Technology of Târgu Mureș, 540139 Târgu Mureș, Romania; lenard.farczadi@umfst.ro; 3 Doctoral School of Medicine and Pharmacy, George Emil Palade University of Medicine, Pharmacy, Science and Technology of Târgu Mureș, 540139 Târgu Mureș, Romania; radupop1982@yahoo.com

**Keywords:** orthodontic anchorage procedures, temporary anchorage devices, bone density, immediate loading, stability

## Abstract

Background: Temporary anchorage devices (TADs) are considered an essential and reliable component of orthodontic fixed appliance therapy, providing stable skeletal anchorage with minimal dependence on patient compliance. Objective: This study aimed to evaluate the success rate of orthodontic mini-implants and to assess the influence of patient- and treatment-related factors on their stability and success rate. Materials and Methods: A retrospective analysis of 60 patients was conducted with a total of 121 inserted TADs. Patient sex, age, orthodontic diagnosis, insertion site, and bone quality were analyzed, and descriptive statistics were calculated for all variables. Associations between categorical variables were evaluated using Pearson’s chi-square test. Variables showing potential association were further analyzed using multivariate binary logistic regression to identify independent predictors of mini-implant success. Results: No statistically significant associations were found between implant success and patient sex (*p* = 0.240), age (*p* = 0.819), or insertion site (*p* = 0.069); however, a trend toward higher failure rates in palatal-placed implants was noted. Logistic regression analysis did not identify any independent predictors of success (*p* = 0.284), and the model explained only a small proportion of outcome variability (5.9–12.0%). Conclusions: The high success rate of the TADs demonstrated high clinical reliability in this study, although patient-related factors such as age and sex did not significantly influence the success rate. Bone quality and soft tissue characteristics may contribute to TAD stability; however, these factors were not directly quantified in the present analysis.

## 1. Introduction

In modern orthodontics, the use of mini-implants as temporary anchorage devices (TADs) is common. Combining these approaches with digital treatment planning can lead to predictable treatment outcomes. Precise anchorage can be obtained by their guided placement, generating controlled forces and tooth movement. To achieve the planned TAD position, surgical guides are required. These can be realized using CAD/CAM techniques based on previous CBCT (cone-beam computed tomography) images. The guided placement helps to avoid anatomical structures during insertion [1,2] and increases efficiency. However, during a single visit, mini-implants and orthodontic devices can be applied simultaneously [3,4].

Most conventional anchorages (headgear, intermaxillary elastics) are conditioned by adjacent teeth or patient compliance, which may negatively affect treatment outcomes [5]. When properly used, orthodontic TADs provide stable skeletal anchorage. They are more aesthetic than extraoral appliances, less dependent on teeth and patient cooperation. Optimal treatment outcomes may be superior to those obtained by conventional techniques [6]. The shorter treatment period and optimal results make these devices an effective alternative for conventional techniques, especially in complex orthodontic cases [7,8,9]. TADs are cost-effective, allowing for an immediate or early loading in a minimally invasive approach. Their stability is only mechanical (without osseointegration) and depends on insertion torque, cortical bone thickness, and bone density [10,11]. A bone thickness of 1 mm or greater is associated with a higher success rate and reduced mini-implant deflection; however, Wilmes et al. recommended a maximum insertion torque of 20 Ncm to prevent mini-implant fracture [12]. Bone quantity and quality, anatomical structures, soft-tissue thickness, and the complexity of the malocclusion may condition TAD placement.

Orthodontic mini-implants differ from prosthodontic implants in both dimensions and biological behavior, typically presenting lengths of 4–12 mm and diameters ranging from 1.15 to 2.5 mm. Unlike conventional dental implants, their surfaces are generally polished, favoring mechanical retention rather than osseointegration. The design components—head, neck, and screw—are selected according to the specific clinical scenario.

The insertion mechanism and overall treatment efficiency are influenced by the type of screw, including self-drilling, self-tapping, thread-forming, and thread-cutting designs [13]. Among these, self-drilling TADs offer the advantage of insertion without the need for predrilling, simplifying the clinical procedure.

According to the classification proposed by Misch, jawbone density can be categorized into four types: D1 (dense cortical bone), D2 (porous cortical bone with coarse trabecular structure), D3 (porous cortical bone with fine trabecular structure), and D4 (fine trabecular bone) [13]. In clinical orthodontic practice, common insertion sites for temporary anchorage devices include the midpalatal region, palatal interdental areas, and buccal interdental regions. These anatomical sites are typically associated with D2 and D3 bone densities, which are considered favorable for achieving adequate primary stability [14].

Midpalatal placement of temporary anchorage devices is associated with high primary stability due to favorable bone quality; however, it may compromise aesthetics. Palatal interdental placement offers improved control of orthodontic forces and tooth movement, while buccal interdental placement is more accessible and, therefore, frequently preferred in clinical practice [14].

The main indications for TAD use include patients with a high-angle maxillary plane, complex orthodontic movements such as intrusion, molar uprighting or distalization, and en masse distalization of the upper or lower dental arches. Additional indications include cases with missing first molars or failure of conventional anchorage methods, particularly in adult patients. Conversely, TAD placement is contraindicated in patients younger than 12 years due to ongoing skeletal development and bone remodeling, as well as in cases presenting pathological bone conditions or diagnosed periodontal disease [13].

The surgical placement of TADs is a minimally invasive procedure, typically performed under local or topical anesthesia, often accompanied by antibiotic prophylaxis [15]. Adequate oral hygiene must be maintained, with careful control of routine tooth brushing [16]. A preoperative chlorhexidine mouth rinse is recommended to reduce microbial load. During insertion, saline irrigation is essential to prevent overheating of the bone and subsequent implant failure. For buccal mini-screws, proper angulation between the roots of the premolars and molars is critical, with recommended insertion angles of 45° to 60° in the maxilla and 10° to 30° in the mandible [17].

Primary stability may be a key element of clinical success [18,19]. According to Selvaraj et al., increased length and a conical thread design can be beneficial [20]. However, their material can influence stability. Orthodontic mini-implants can be made from stainless steel or from four types (I–IV) of decreasing-purity titanium alloys. The most popular titanium alloy used for their manufacturing is type V (Ti6Al4V), which contains 6% aluminum and 4% vanadium. This type has good mechanical properties and corrosion resistance [21]. Its bioactivity is lower than that of pure titanium, and its osteointegration capacity is lower. Attentive biomechanical principles are considered each time anchorage is provided with TADs, especially when they are loaded immediately after placement. Several studies have reported that immediate loading may have a beneficial effect on peri-implant bone quality [22]; however, other authors have suggested delayed loading periods ranging from 1 to 6 weeks [23].

According to Motoyoshi et al., TADs are considered successful if they remain clinically stable and functionally effective throughout their use as anchorage, without exhibiting mobility, significant inflammation, or premature loss [23].

Despite the widespread use of orthodontic mini-implants and the substantial body of literature supporting their clinical effectiveness, the factors influencing their success remain incompletely understood. Previous studies have reported variable outcomes regarding the impact of patient-related variables, insertion site, and biomechanical conditions on implant stability, often with inconsistent or contradictory results. Moreover, the multifactorial nature of TAD success, involving bone quality, soft tissue characteristics, insertion technique, and loading protocols, highlights the need for further investigation. Therefore, a comprehensive evaluation of these influencing factors is essential to better understand their relative contribution to clinical outcomes and to optimize treatment protocols.

In this context, the present study aimed to evaluate the stability and success rate of immediately loaded orthodontic TADs and to investigate the influence of patient- and treatment-related factors, such as sex, age, orthodontic diagnosis, and bone quality at the insertion site, on their clinical performance.

## 2. Materials and Methods

The study protocol was approved by the Institutional Ethics Committee of George Emil Palade University of Medicine, Pharmacy, Sciences and Technology of Târgu Mureș (approval number 4055/20.03.2026). The reporting of this retrospective observational study was performed in accordance with the STROBE (Strengthening the Reporting of Observational Studies in Epidemiology) guidelines.

**Figure 1:**
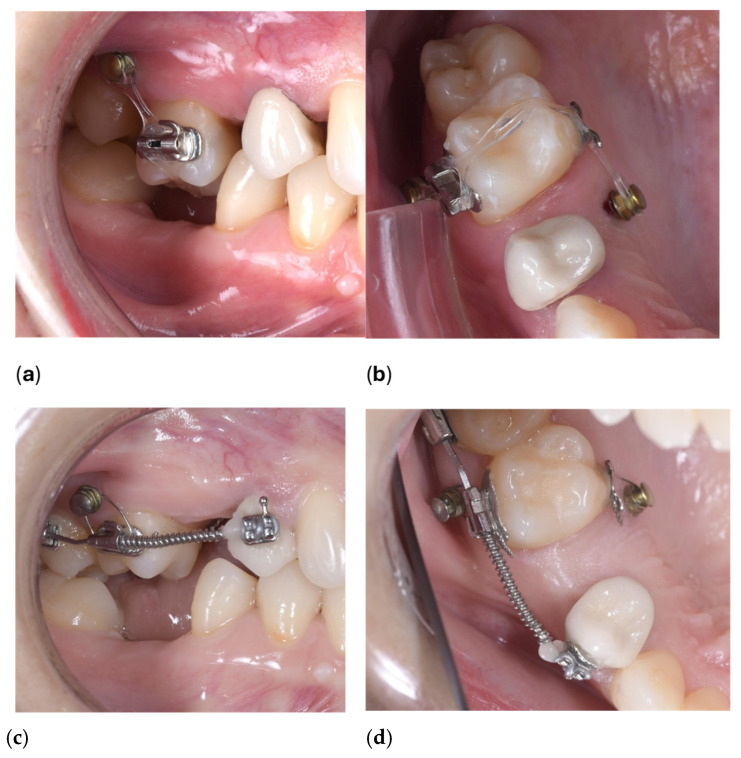
Upper molar intrusion with TADs: (**a**,**b**) immediately after placement and loading (**c**,**d**) after intrusion was completed.

Patients included in this retrospective study were selected from the clinical database of the Department of Orthodontics, George Emil Palade University of Medicine, Pharmacy, Sciences and Technology of Târgu Mureș, where all treatments were performed according to standardized clinical protocols. The inclusion criteria were as follows: (1) patients treated with fixed orthodontic appliances between January 2022 and December 2025; (2) patients who completed orthodontic treatment or had documented TAD follow-up; (3) use of at least one immediately loaded temporary anchorage device; (4) complete clinical and radiographic documentation available with information regarding the TAD (e.g., insertion site, TAD characteristics). Patients with incomplete documentation, interrupted orthodontic treatment, missing follow-up data, poor oral hygiene and were younger than 12 years were excluded. A screening of patients’ records was performed to identify patients who underwent orthodontic treatment between January 2022 and December 2025, and cases in which orthodontic TADs were used for anchorage enforcement were selected. Cases with at least one inserted TAD and complete clinical records (diagnostics, implant parameters, insertion site, and treatment outcomes) were selected to achieve consistency in data collection and minimize variability. Figure 1a–d shows the clinical use of TADs inserted vestibularly and palatally for molar intrusion.

The study group consisted of 60 patients (14 males and 46 females, mean age: 29 ± 16 years) with a total of 121 inserted TADs (PSM Quatro Mini RH, PSM, North America, Inc., La Quinta, CA, USA). All the inserted TADs were self-drilling with a perforated head and a conical body with a diameter of 1.5 mm at the neck and 1.4 mm at the tip. The length of the TADs was selected according to the insertion site: palatally placed TADs were 9–10 mm, upper labially placed TADs were 8–9 mm, and lower labially placed ones were 6–7 mm in length. TADs were placed in three insertion directions: 40 in the palatal area, 45 in the upper vestibular area, and 36 in the lower vestibular region (Table 1). The distribution of TADs was examined in four anatomical regions (incisors, canine-first bicuspid (C–PM1), between the two bicuspids (PM2–M1), and between first and second molars (M1–M2)).

TAD success was defined as the maintenance of clinical stability throughout orthodontic treatment, without clinically detectable mobility, pain, peri-implant inflammation, or premature loss under orthodontic loading. Failure was defined as clinically detectable mobility, inflammation-associated loosening, or loss of the TAD before completion of the intended orthodontic tooth movement.

Clinical evaluation was performed during routine orthodontic follow-up visits, including assessment of implant stability, peri-implant soft tissue condition, and signs of inflammation.

Bone quality at the insertion site was assessed retrospectively using clinical records and radiographic evaluation based on the Misch bone density classification system (D1–D4). Anatomical insertion regions and cortical bone characteristics were evaluated using pre-treatment radiographs and CBCT images when available.

The data were recorded and processed in Microsoft Excel; the following variables were analyzed: patient characteristics (sex, age, and orthodontic diagnosis) and bone quality at the insertion site. Statistical analysis was performed using IBM SPSS Statistics version 25 (IBM Corp., Armonk, NY, USA). Descriptive statistics were calculated for all variables. Associations between categorical variables were evaluated using Pearson’s chi-square test. Variables showing potential association were further analyzed using multivariate binary logistic regression to identify independent predictors of mini-implant success. Odds ratios (ORs) with 95% confidence intervals (CIs) were calculated. A *p*-value < 0.05 was considered statistically significant. A post hoc power analysis was conducted using G*Power 3.1.9.7 (Heinrich-Heine-Universität, Düsseldorf, Germany) based on a total sample size of 121 participants.

## 3. Results

The statistical analysis performed in this study aimed to evaluate the stability and success of immediately loaded, identical-shaped orthodontic TADs and to determine whether certain patient- and treatment-related factors (patient sex, age, orthodontic diagnosis, bone quality at the insertion site) can influence this stability.

### 3.1. Influence of Patient Sex and Age on TAD Success

The female:male ratio at initial insertion was 3.16:1; 39 TADs out of the total of 121 were inserted in the mandible, while 82 out of 121 were inserted in the maxilla.

The Chi-square Pearson analysis showed that patient sex was not significantly associated with micro-implant success (χ^2^(1) = 1.38, *p* = 0.240). The failure rate appeared slightly higher in male patients (7 failures out of 47) than in females (6 failures out of 74), but the difference was not statistically significant. Therefore, gender cannot be considered a determining factor for TAD stability in this sample.

Patient age did not show a statistically significant relationship with micro-implant success (χ^2^(3) = 0.92, *p* = 0.819). More than 80% of TADs were placed in patients over 20 years of age, but the success rate was identical in subjects from the adult group and those under 19 years of age. Based on our results, we believe that age alone will not influence the success rate and stability of TADs.

### 3.2. Influence of Malocclusion on TAD Success

Complex diagnosis was found in most of the cases and the division of TADs by malocclusion was as follows: most of the TADs were used in cases with frontal crowding (34 TADs out of 121 (28%)), followed by deep bite (31 TADs out of 121 (25.61%)) and Angle Class II Division 1 malocclusion (29 TADs out of 121 (23.96%)). In cases of increased overjet (18 TADs out of 121 (14.87%)), anterior open bite (10 TADs out of 121 (8.26%)), Angle Class II Division 2 malocclusion (8 TADs out of 121 (6.60%)), and posterior crossbite (7 TADs out of 121 (5.78%)), the TADs were used less often, while anterior crossbite showed the lowest frequency (2 TADs out of 121 (1.65%)). The chi-square test showed a significant difference in the distribution according to diagnoses (χ^2^ = 390.2, df = 7, *p* < 0.001), which means that the use of TADs is not uniform and significantly depends on the type of malocclusion.

### 3.3. Influence of Insertion Site on TAD Success

The results showed that the largest number of TADs were inserted at the PM2–M1 level in all three sites (superior vestibular: *n* = 17; palatal: *n* = 18; inferior vestibular: *n* = 16). This region showed the highest values across all categories, and its distribution can be considered relatively homogeneous (Table 2).

In the incisor region, only superior labial insertion was performed (*n* = 15), while no insertion was performed in the palatal and inferior labial directions (*n* = 0–0), indicating a strongly asymmetric distribution. A more balanced distribution was observed in the C–PM1 region (superior vestibular: *n* = 8; palatal: *n* = 8; inferior vestibular: *n* = 11), with a slight dominance of the inferior vestibular region. In the M1-M2 region, palatal insertion predominated (*n* = 14), while superior vestibular (*n* = 5) and inferior vestibular (*n* = 9) showed a lower frequency. The data suggest that the distribution is not uniform across insertion sites and directions, and that certain regions (especially PM2-M1) play a prominent role (Table 3).

The chi-square test showed a significant relationship between the location and direction of mini-screw insertion (χ^2^ = 32.53; df = 6; *p* < 0.001). This means that the distribution of TADs is not random; there is a statistically significant relationship between the insertion region and direction.

The largest deviation from the expected values was observed in the incisal region, where only the upper vestibular insertion was performed, while in other regions, a more balanced distribution was present. The PM2-M1 region showed high and almost identical values in all directions, which supports its clinical preference.

In the binary logistic regression analysis, we examined the relationship between the success of mini screws and the region of insertion (Table 4). Palatal insertion was chosen as the reference category. Based on the results, the chance of success for mini screws placed in the upper vestibular region was 3.67 times higher (OR = 3.67; 95% CI: 0.90–14.9; *p* = 0.07), while the chance of success in the lower vestibular region was 4.48 times higher (OR = 4.48; 95% CI: 0.95–21.2; *p* = 0.06) compared to the palatal region.

The Hosmer–Lemeshow test did not show a significant difference (*p* = 0.83), indicating a good fit of the model. Although the differences were not statistically significant, the obtained odds ratios show a clinically relevant trend that mini screws placed in vestibular regions may achieve a higher success rate.

A binary logistic regression was conducted to examine whether sex, age, and insertion site predicted TAD success. The overall model was not statistically significant (χ^2^(6) = 7.42, *p* = 0.284), indicating that the predictors did not reliably distinguish between successful and unsuccessful cases. The model explained between 5.9% (Cox and Snell R^2^) and 12.0% (Nagelkerke R^2^) of the variance in success.

The Hosmer–Lemeshow goodness-of-fit test was non-significant (χ^2^(8) = 6.86, *p* = 0.552), suggesting acceptable model fit. However, none of the individual predictors were statistically significant.

Sex was not associated with success (OR = 1.83, 95% CI [0.52, 6.49], *p* = 0.347), and age was not significant overall (*p* = 0.647). Post hoc power analysis indicated that the sample size of 121 provided 72% power to detect this effect size at α = 0.05.

The insertion site showed a non-significant trend (*p* = 0.072). Overall classification accuracy was 89.3%, but the model failed to correctly classify any unsuccessful cases, indicating limited practical predictive value. This indicates that the model mainly predicted the dominant outcome (success) and therefore has limited practical predictive value for identifying potential failures.

**Figure 2:**
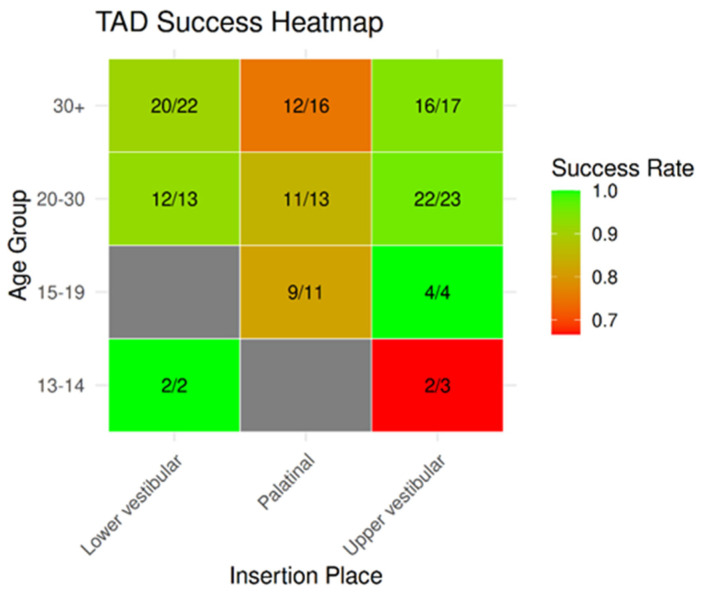
Success rates of orthodontic TADs by age group and insertion site.

**Figure 3:**
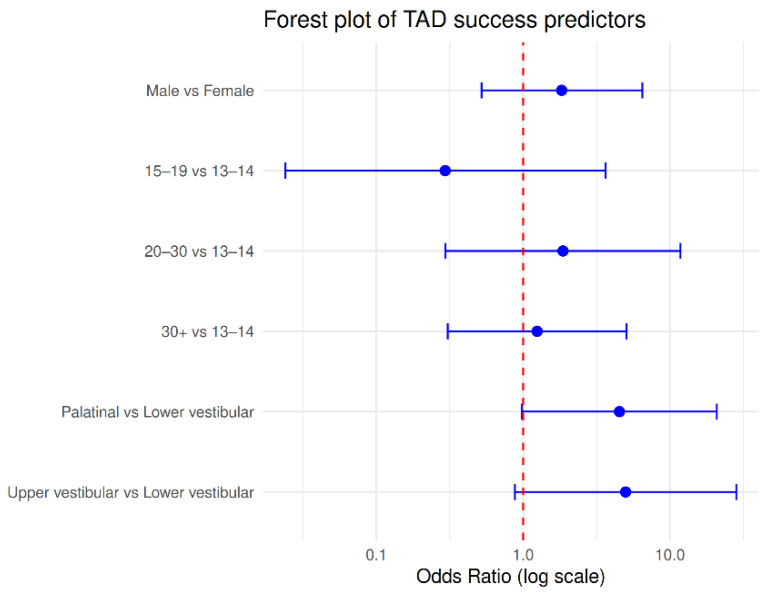
Odds ratios (ORs) and 95% confidence intervals (CIs) for orthodontic micro-implant (TAD) success by patient sex, age group, and insertion site.

To visualize TAD success, two complementary figures were generated in R (version 4.3.1; R Foundation for Statistical Computing, Vienna, Austria) using the ggplot2 package. A heatmap was created to show success rates across age groups and insertion sites, with each tile colored according to the proportion of successful TADs and annotated as Success/Total (Figure 2). Missing combinations of age and insertion site were included as empty cells to ensure that all possible groups were represented. Additionally, a Forest plot was constructed to display odds ratios (ORs) and 95% confidence intervals (CIs) from a multivariable binary logistic regression assessing the effects of sex, age group, and insertion site on TAD success. CIs crossing 1.0 indicate non-significant predictors (Figure 3).

When comparing patients’ gender, age, insertion site, and malocclusion, the multivariate logistic regression model was not significant (χ^2^(6) = 7.42; *p* = 0.284), suggesting that the variables examined together are not able to reliably predict success. The low explanatory power of the model (Nagelkerke R^2^ = 12%) further reinforces this.

Although a non-significant trend was observed for insertion site (*p* = 0.072), neither gender nor age showed a significant effect. Despite the high classification accuracy of the model (89.3%), it was unable to identify unsuccessful cases, which limits its clinical applicability.

## 4. Discussion

### 4.1. Principal Findings

The present retrospective study evaluated the success rate of immediately loaded orthodontic temporary anchorage devices (TADs) and investigated the influence of patient- and treatment-related factors on their clinical performance. The overall success rate was high, confirming the reliability of immediately loaded TADs as a skeletal anchorage method in orthodontic treatment. Neither patient sex nor age emerged as significant predictors of TAD success. Similarly, insertion sites were not significantly associated with implant survival, although a non-significant trend toward higher success rates in vestibular insertion sites was observed. Furthermore, all failures occurred in D3 bone, suggesting that local anatomical characteristics may contribute to primary stability.

### 4.2. Overall Success Rate and Comparison with Previous Studies

The high success rate observed in the present study is consistent with previous investigations reporting success rates ranging from 80% to 95% for orthodontic mini-implants [24,25]. These findings support the concept that immediately loaded TADs represent a predictable and reliable treatment modality for achieving skeletal anchorage while minimizing dependence on patient compliance.

The relatively low number of failures in our sample may be explained by the standardized clinical protocol used for TAD placement, the use of self-drilling implants, and the fact that all procedures were performed by the same experienced operator. Similar observations have been reported in previous studies, suggesting that careful case selection and standardized insertion techniques contribute substantially to implant stability [26,27].

### 4.3. Influence of Patient-Related Factors

Patient sex was not significantly associated with TAD success. Although a slightly higher failure rate was observed among male patients, the difference was not statistically significant. These findings are in agreement with previous studies that reported no significant influence of sex on orthodontic mini-implant stability [28,29,30].

Similarly, chronological age was not associated with TAD success. Although most TADs were placed in patients older than 20 years, the success rates were comparable between younger and older individuals. Previous studies have also demonstrated that age alone is not a reliable predictor of mini-implant stability [31,32,33,34].

The absence of an age effect may be explained by the fact that local anatomical characteristics, particularly cortical bone thickness and bone density at the insertion site, are likely more important determinants of primary stability than chronological age itself. Consequently, clinicians should focus on individual anatomical conditions rather than age when selecting insertion sites and planning treatment protocols [35,36,37].

### 4.4. Influence of Insertion Site and Bone Quality

Although insertion site did not significantly influence TAD success, a clinically relevant trend toward higher success rates in vestibular regions was observed. The odds of success were approximately four times higher for vestibular placements than for palatal placements, although these differences did not reach statistical significance.

The absence of statistical significance may be attributable to the relatively small sample size and the limited number of failed cases, resulting in wide confidence intervals. Nevertheless, the observed trend suggests that anatomical location may influence TAD stability. Differences in cortical bone thickness, soft tissue characteristics, and biomechanical loading conditions between palatal and vestibular regions may partially explain these findings [5,38,39].

The PM2–M1 region represented the most frequently used insertion site in the present study, indicating its favorable biomechanical characteristics and accessibility. The non-uniform distribution of insertion sites further suggests that TAD placement is largely determined by clinical and anatomical considerations rather than by random selection [15].

Bone quality also appeared to influence implant stability. All failures occurred in D3 bone, whereas no failures were observed in D1 or D2 bone. These findings are consistent with previous reports emphasizing the importance of cortical bone thickness and bone density for achieving adequate primary stability [4,6,40]. Since orthodontic mini-implants rely primarily on mechanical retention rather than osseointegration, local bone characteristics may play a decisive role in determining clinical outcomes.

In clinical situations involving lower-density bone, strategies such as selecting longer or wider implants, optimizing insertion angulation, or reducing immediate loading forces may improve implant stability and reduce the risk of failure.

### 4.5. Clinical Implications

The present findings suggest that immediately loaded TADs can be used successfully in a broad range of orthodontic patients when appropriate case selection and standardized insertion protocols are applied. Although patient-related factors such as age and sex do not appear to significantly influence outcomes, clinicians should pay particular attention to local anatomical conditions, including bone quality and soft tissue characteristics.

The observed tendency toward higher success rates in vestibular insertion sites may be clinically relevant when selecting the most appropriate anchorage location. Furthermore, knowledge of bone density characteristics may facilitate individualized treatment planning and improve the predictability of orthodontic anchorage.

### 4.6. Study Limitations and Future Directions

Several limitations should be considered when interpreting the results of this study. First, despite the inclusion of 121 TADs, these implants originated from only 60 patients, and multiple implants were inserted in some individuals. Consequently, the observations cannot be considered completely independent.

Second, the relatively small number of failures limited the statistical power of the regression analyses and prevented the application of more advanced mixed-effects models. Therefore, the regression findings should be interpreted as exploratory and hypothesis-generating.

Third, the retrospective design limited control over potential confounding variables. Important factors such as insertion torque, cortical bone thickness, peri-implant soft tissue characteristics, oral hygiene, and biomechanical loading conditions were not directly evaluated and may have influenced implant stability.

Finally, although all procedures were performed by the same experienced orthodontist, which reduced operator variability, this may limit the generalizability of the findings to other clinical settings.

Future prospective multicenter studies with larger sample sizes are required to simultaneously evaluate patient-related, anatomical, and biomechanical factors influencing TAD success. The integration of three-dimensional imaging, digital treatment planning, and artificial intelligence-based predictive models may further improve the understanding of TAD stability and support personalized orthodontic treatment planning.

## 5. Conclusions

Overall, the results of the study support that the insertion site of TADs not only influences the frequency of use, but also potentially the success rate and clinical outcome; further prospective studies with larger sample sizes are needed to confirm the results. Vestibular insertion sites demonstrated a tendency toward higher success rates compared with palatal insertion; however, these findings were not statistically significant and should be interpreted cautiously. Patient-related factors such as age and sex, as well as insertion site, did not significantly influence TAD stability and success rate.

Bone quality and soft tissue characteristics may contribute to TAD stability; however, these variables were not directly quantified in the present analysis. Placement in keratinized, attached gingiva and areas of higher bone density was associated with more favorable outcomes, whereas insertion in porosus compact bone or mobile, non-keratinized mucosa was linked to increased risk of failure.

The PM2-M1 region seems to be the more stable one, while the distribution by direction shows region-dependent variability. The need for vertical control and stable skeletal anchorage demands the use of TADs for anchorage enforcement, especially in deep-bite and frontal crowding cases.

Given the limited sample size and retrospective design, further prospective studies using larger samples and multivariate models are required to study TADs’ additional biomechanical parameters to better identify factors that can influence TADs’ success rate and stability, and to optimize clinical protocols.

## Figures and Tables

**Table 1:** Failure and success rate within the study group by gender, age, and insertion site.

Gender	Age	Insertion Site	Number	Succes	Failure
Female	13–14 year	Upper buccal	1	1	0
		Lower buccal	1	1	0
	15–19 year	Palatal	3	2	1
		Upper buccal	1	1	0
	20–30 year	Palatal	11	9	2
		Upper buccal	16	16	0
		Lower buccal	8	7	1
	30+ year	Palatal	8	6	2
		Upper buccal	11	11	0
		Lower Buccal	14	14	0
Male	13–14 year	Upper buccal	2	2	0
		Lower buccal	1	1	0
	15–19 year	Palatal	8	7	1
		Upper buccal	3	3	0
	20–30 year	Palatal	2	2	0
		Upper buccal	7	6	1
		Lower buccal	5	5	0
	30+ year	Palatal	8	6	2
		Upper buccal	6	5	1
		Lower Buccal	9	8	1

**Table 2:** Success rates of orthodontic TADs by insertion site.

Insertion Site	Upper Vestibular	Palatal	Lower Vestibular	Total
Incisor	15	0	0	15
C-PM1	8	8	11	27
PM2-M1	17	18	16	51
M1-M2	5	14	9	28
Total	45	40	36	121

**Table 3:** Success rates of orthodontic TADs by insertion region and site.

Insertion Site	Total Insertions	Successful Insertions	Succes Rate
Upper vestibular	47	44	93.63%
Palatal	40	32	80.00%
Lower Vestibular	38	36	94.74%

**Table 4:** Distribution and clinical outcome of TADs according to Misch bone quality classification.

Misch Bone Quality	Number of TADs (*n*)	Successful	Failed
D1	6	6	0
D2	11	11	0
D3	104	91	13
D4	0	0	0
Total	121	108	13

## Data Availability

The datasets generated and analyzed during the current study are available from the corresponding author upon reasonable request.

## Abbreviations

The following abbreviations are used in this manuscript:

TAD	Temporary Anchorage Device
PM1	First premolar
PM2	Second premolar
M1	First Molar
